# The transcription of the alarmin cytokine interleukin-1 alpha is controlled by hypoxia inducible factors 1 and 2 alpha in hypoxic cells

**DOI:** 10.3389/fimmu.2012.00290

**Published:** 2012-09-14

**Authors:** Peleg Rider, Irena Kaplanov, Marianna Romzova, Liora Bernardis, Alex Braiman, Elena Voronov, Ron N. Apte

**Affiliations:** The Shraga Segal Department of Microbiology and Immunology and The Cancer Research Center, Faculty of Health Sciences, Ben-Gurion University of the NegevBeer-Sheva, Israel

**Keywords:** alarmin, IL-1, sterile inflammation, HIF-1α, HIF-2, DAMPs, cytokines and inflammation

## Abstract

During hypoxia, cells undergo transcriptional changes to adjust to metabolic stress, to promote cell survival, and to induce pro-angiogenic factors. Hypoxia-induced factors (HIFs) regulate these transcriptional alterations. Failure to restore oxygen levels results in cell death by necrosis. IL-1α is one of the most important mediators of sterile inflammation following hypoxia-mediated necrosis. During hypoxia, IL-1α is up-regulated and released from necrotic cells, promoting the initiation of sterile inflammation. This study examined the role of IL-1α transcription in initiation of hypoxic stress and the correlation between IL-1α transcription and HIFα factors. In an epithelial cell line cultured under hypoxic conditions, IL-1α transcription was up-regulated in a process mediated and promoted by HIFα factors. IL-1α transcription was also up-regulated in hypoxia in a fibroblast cell line, however, in these cells, HIFα factors inhibited the elevation of transcription. These data suggest that HIFα factors play a significant role in initiating sterile inflammation by controlling IL-1α transcription during hypoxia in a differential manner, depending on the cell type.

## INTRODUCTION

During infections, injuries, infarcts, or other ischemic events, tissue cells experience hypoxic stress, which can result in cell necrosis that induces inflammation. In infectious diseases, in addition to molecules originating in necrotic tissue, bacterial products alert the immune system following pattern recognition. However, dying cells in a sterile environment use self-molecules alone to signal the surrounding cells and the immune system of the danger which confronts the tissue (Matzinger, 1994). Among the cell molecules released from necrotic cells, some are inducers of sterile inflammation, and were termed “alarmins” or “danger-associated molecular patterns” (DAMPs; Oppenheim and Yang, 2005; Bianchi, 2007; Rubartelli and Lotze, 2007; Chen and Nunez, 2010). The immune response to dead cells includes myeloid cell recruitment. These cells, mostly granulocytes and macrophages, migrate to the hypoxic area, counter to the oxygen gradient (Lotfi et al., 2009; Eltzschig and Carmeliet, 2011), where they can promote debris clearance and tissue repair or a pro-angiogenic response (Nizet and Johnson, 2009). Alternatively, myeloid cells can expand the inflammatory response which can lead to additional tissue damage. Several alarmin molecules have been described. Among these are HMGB1, S100 proteins, heat-shock proteins, and IL-33 (Hofmann et al., 1999; Basu et al., 2000; Raucci et al., 2007; Moussion et al., 2008; Chen and Nunez, 2010; Andersson and Tracey, 2011). IL-1α is a major alarmin molecule that was shown to be a key inducer of sterile inflammation (Chen et al., 2007; Eigenbrod et al., 2008; Cohen et al., 2010; Dinarello et al., 2012). In addition to its alarmin property in response to dying cells, IL-1α can also differentiate between apoptosis and necrosis, by its restricted release from necrotizing but not apoptotic cells (Luheshi et al., 2009; Cohen et al., 2010).

During hypoxia, cells alter their transcriptome in order to adjust to changes in the availability of oxygen and to metabolic stress. The alteration in transcription is mediated by hypoxia-induced factors (HIFs), which are heterodimer transcription factors, composed of a stable beta subunit and an alpha subunit that has a half-life of several minutes. The alpha subunit is targeted for the ubiquitin proteasome degradation pathway, as long as its specific proline residues are hydroxylated by prolyl hydroxylases (PHDs; Bruick and McKnight, 2001), which allows recognition by von Hippel–Lindau (VHL) ubiquitin E3-ligase (Jaakkola et al., 2001). HIFα proteins include HIF-1 to -3, while HIF-1α and HIF-2α are considered to be the major HIF transcription factors that control vast gene transcription and can have differential or even opposing effects (Hu et al., 2003; Wang et al., 2005; Keith et al., 2012). HIF-1α and HIF-2α correlated not only with cell metabolism and angiogenesis accompanied by hypoxia, but also with the inflammatory response during infection or tissue damage (Nizet and Johnson, 2009).

It was shown that during hypoxia, macrophage secretion of IL-1α increased in conjunction with pro-angiogenic factors such as VEGF (Carmi et al., 2009). While bone marrow-derived cells can respond to tissue stress or infection by secreting many pro-inflammatory cytokines, chemokines, proteases, reactive oxygen species (ROS), etc., non-hematopoietic tissue resident cells can also induce inflammation, but in a more restricted manner, by secreting IL-1α, one of the most potent cytokines found in these cells. Among such cells are fibroblasts (Kawaguchi et al., 2006), keratinocytes (Kong et al., 2006; Lee et al., 2009; Rider et al., 2011), endothelial cells (Berda-Haddad et al., 2011), and hepatocytes (Sakurai et al., 2008; Kamari et al., 2011). Recently, we showed that hypoxic cell-derived IL-1α induces inflammation in Matrigel plugs. IL-1α was up-regulated in hypoxic cells which eventually died by necrosis. The up-regulation was observed both on the mRNA and protein levels in keratinocytes cells. However, whether HIFα proteins where involved in this up-regulation was not yet studied. The up-regulation and release of IL-1α mediated an influx of neutrophils in early stage, followed by macrophage infiltration, which was an IL-1β-dependent phase of the inflammatory process (Rider et al., 2011). Thus, in the present study, we examined the role of major hypoxic transcription factors, the HIFα proteins, on the initiation of the transcription and regulation of IL-1α, the alarm cytokine, which characterizes sterile inflammation. A link between HIFα regulation and the elevation of IL-1α can add a new functional role for the HIFα proteins, as regulators of sterile inflammation, which when chronic local angiogenesis is switched on.

## MATERIALS AND METHODS

### CELL CULTURE

WI-38, A549, HeLa, and HEK-T293 cells were cultured in DMEM (Invitrogen, Carlsbad, CA, USA), supplemented with 10% heat-inactivated fetal bovine serum (FBS), 2mM l-glutamine, 100 U/ml penicillin, and 100 μg/ml streptomycin (Biological Industries, Beit Haemek, Israel). For hypoxic stress, cells were cultured in a sealed anaerobic workstation (Concept 400; Ruskinn Technology/Jouan) providing conditions of O_2_< 0.3%, 5% CO_2_, 95% N_2_, and 37°C.

### siRNA SILENCING

Cells were transfected using the jetPRIME transfection reagent (Polyplus transfection), according to the manufacturer’s instructions, with 100 nM of non-targeting pool control siRNA, on-target plus SMARTpool human HIF1A (3091) or EPAS1 (2034), all from Thermo Scientific.

### VECTORS AND TRANSFECTIONS

A549, WI-38, and HeLa cells were transfected using the JetPEI reagent (Polyplus transfection), while HEK-T293 were transfected using the calcium-phosphate method as described before (Rider et al., 2011), with plasmids encoding HA-HIF-1α-P402A/P564A and HA-HIF-2α-P405A/P531A mutated sequences which were previously described (Kondo et al., 2003), and were a gift from Professor William Kaelin (addgene plasmid #18955 and #18956). In order to obtain a control plasmid, the insert of HA-HIF-2α-P402A/P564A vector, was liberated with *Bam*HI and *Not*I restriction enzymes, overhang ends were filled with DNA polymerase I large (Klenow) fragment enzyme and ligated to obtain control circular plasmid encoding HA with no HIFα insert. All enzymes in this procedure were from New England Biolabs.

### WESTERN BLOT

Nuclear and cytosol fractions of HEK-T293 transfected cells were prepared with NE-PER Nuclear and cytoplasmic extraction reagents (Thermo Scientific). Nuclear fractions were separated over 8% PAGE and transferred to PVDF membranes (Millipore). Detection of HIFα proteins was performed using mouse anti-HIF-1α (Novus Biological) and rabbit anti-HIF-2α (Abcam) antibodies. To detect IL-1α, cells were centrifuged and pellets were re-suspended in 0.5% Triton-X100 in PBS supplemented with protease inhibitor cocktail (Calbiochem). Lysates were centrifuged and protein concentrations were calculated using the Bradford reagent (Bio-Rad). Lysates were separated over 15% PAGE, and IL-1α was detected on PVDF membranes using mouse anti-IL-1α antibodies (R&D).

### QUANTITATIVE RT-PCR

Total RNA was extracted from the cells using RNeasy kit (Qiagen, Valencia, CA, USA), and quantified using a NanoDrop spectrophotometer (ND-1000 spectrophotometer, NanoDrop Technologies, USA). cDNA reverse-transcription was performed with 1 μg of total RNA as a template, using the qScript cDNA Synthesis Kit (Quanta Biosciences). The quantitative RT-PCR was performed with PerfeCta SYBR Green FastMix, Low ROX (Quanta Biosciences) on ABI Prism 7500 sequence detection system (Applied Biosystems). In house SYBR Green based assays were used to quantify human β-actin: AGCCTCGCCTTTGCCGATCC, TTGCACATGCCGGAGCCGTT; IL-1α: GCCCAAGATGAAGACCAACCAGTGC, GCCGTGAGTTTCCCAGAAGAAGAGG; VEGF: CTACCTCCACCATGCCAAGTGGTCC, ATGTCCACCAGGGTCTCGATTGGA; HIF-1α: AGACTTTCCTCAGTCGACACAGCCT, GCGGCCTAAAAGTTCTTCTGGCTCA; and EPAS1: TGCTCCACGCCCAATAGCCC, GGGTGCCAGTGTCTCCAA-GTCC.

Relative quantification was calculated by the 2^ΔΔCq method. Averages of ΔCq from biological replicates or from different experiments were analyzed by two-tailed Student’s *t*-test for statistical significance using GraphPad Prism 4 (GraphPad Software).

## RESULTS

### IL-1α TRANSCRIPTION IS UP-REGULATED DURING HYPOXIA IN THE HUMAN EPITHELIAL CELL LINE A549

**FIGURE 1:**
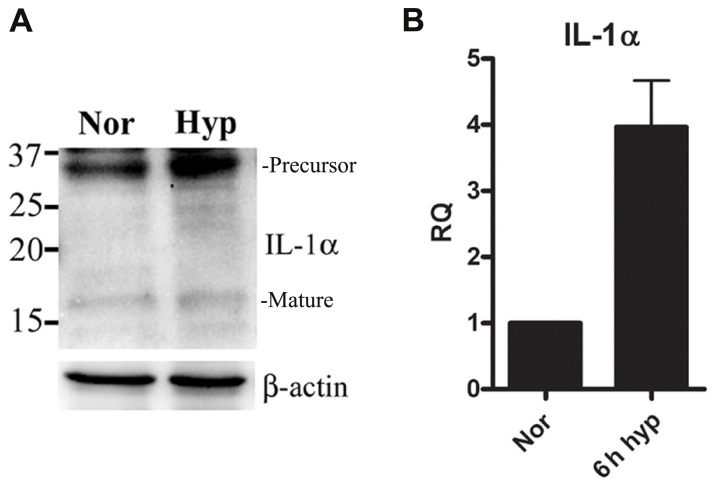
**IL-1α transcription is up-regulated during hypoxia in A549 epithelial cells.(A)** A549 cells were cultured in either normoxic or hypoxic conditions for 12 h. Cells lysates were analyzed by Western blot for IL-1α detection. Bands are marked as precursor or mature. **(B)** A549 cells were cultured in either normoxic or hypoxic conditions for 6 h, and were analyzed for relative quantification (RQ) of IL-1α transcription. Graph represents mean ± SD of three biological repeats.

We recently reported that IL-1α is up-regulated in mouse keratinocytes during hypoxia (Rider et al., 2011). This up-regulation of IL-1α together with the accompanied necrosis following extended periods of hypoxia (24 h) resulted in increased levels of IL-1α in the cell supernatants. Therefore, we sought to elucidate the initial steps of up-regulation of IL-1α transcription before cells are damaged due to acidosis and necrosis. We cultured the lung epithelial A549 cell line in either normal or hypoxic conditions, and detected up-regulation of the 31 kDa precursor protein and to some extent the 17 kDa mature cytokine (**Figure 1A**), similar to data with mouse keratinocytes reported in our recent paper (Rider et al., 2011). Since our goal was to study the initial phase of IL-1α up-regulation, we evaluated mRNA levels during hypoxia. Cells were cultured in a hypoxic chamber for 6 h and then analyzed for IL-1α transcription by real-time PCR. Indeed, IL-1α transcription during the initial phase of hypoxia was up-regulated compared to cells cultured in normoxia (**Figure 1B**).

### SILENCING HIFα PROTEINS DURING HYPOXIA RESULTS IN ATTENUATED IL-1α TRANSCRIPTION

**FIGURE 2:**
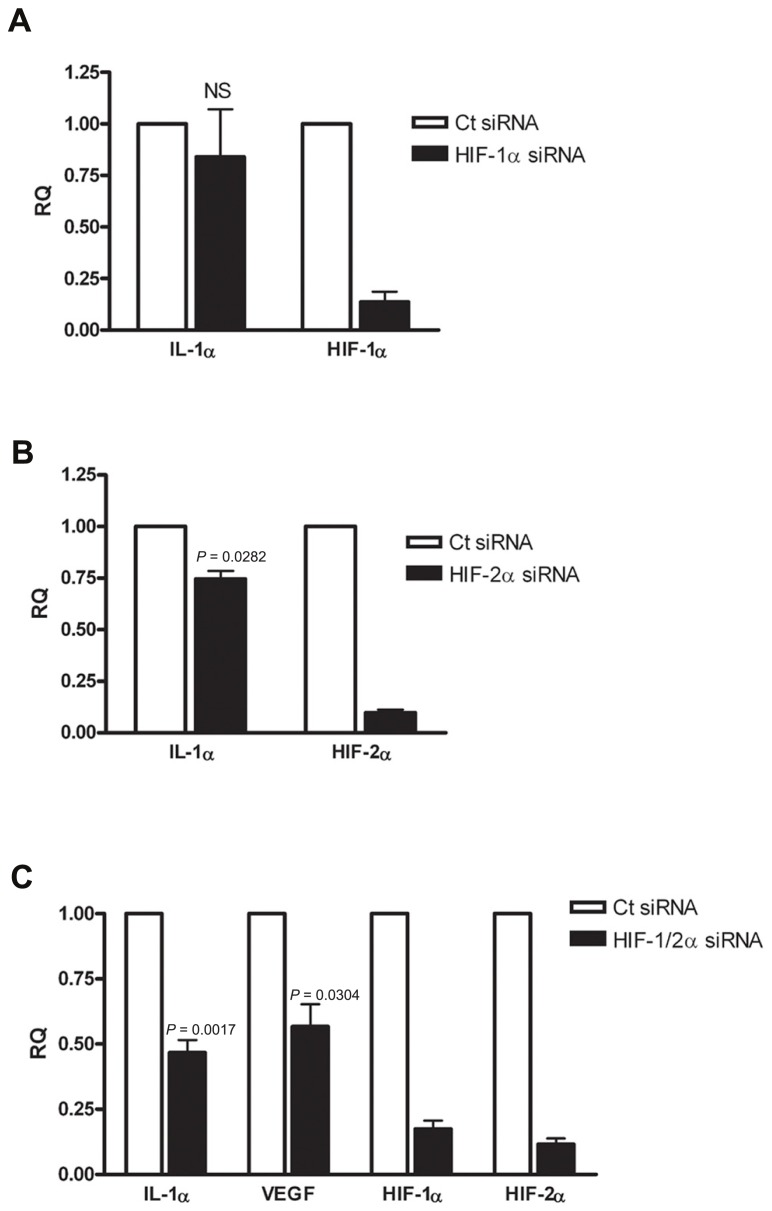
**Silencing HIFα proteins down-regulates IL-1α transcription in hypoxic A549 epithelial cells.** A549 cells were transfected with HIF-1α siRNA **(A)** or HIF-2α siRNA **(B)** 24 h prior to hypoxia (6 h). Each graph represents mean ± SEM of three independent experiments. **(C)** Cells were transfected with both HIF-1α and HIF-2α mixed siRNA 24 h prior to hypoxia (6 h). Graph represents mean ± SEM of five independent experiments. *P* values of HIF-2α or HIF-1/2α siRNA-transfected cells vs. control (Ct) siRNA-transfected cells are annotated in the graphs.

Since IL-1α transcription was altered during hypoxia, we examined whether HIFα proteins are involved in IL-1α up-regulation. First, we assessed HIF-1α siRNA silencing, and no significant change in IL-1α transcription was observed (**Figure 2A**). Levels of HIF-1α were also measured in order to assure that silencing was successful. Next, we examined whether silencing of the other major HIFα transcription factor, HIF-2α, altered the transcription of IL-1α. We observed that HIF-2α silencing resulted in a minor reduction of IL-1α mRNA levels (**Figure 2B**). However, the use of both HIF-1α and HIF-2α as targets for siRNA silencing resulted in significantly attenuated levels of IL-1α mRNA in A549 cells, similar to that of VEGF (**Figure 2C**). These data indicates that HIFα factors promote the up-regulation of IL-1α during hypoxia and increase the inflammatory potential in cases of hypoxia-mediated necrosis. In addition, silencing HIFα factors in order to decrease angiogenesis, for example, by inhibiting VEGF transcription, can result in decreased levels of IL-1α as well.

### SILENCING HIFα PROTEINS DURING HYPOXIA RESULTS IN INCREASED IL-1α TRANSCRIPTION IN WI-38 LUNG FIBROBLASTS

**FIGURE 3:**
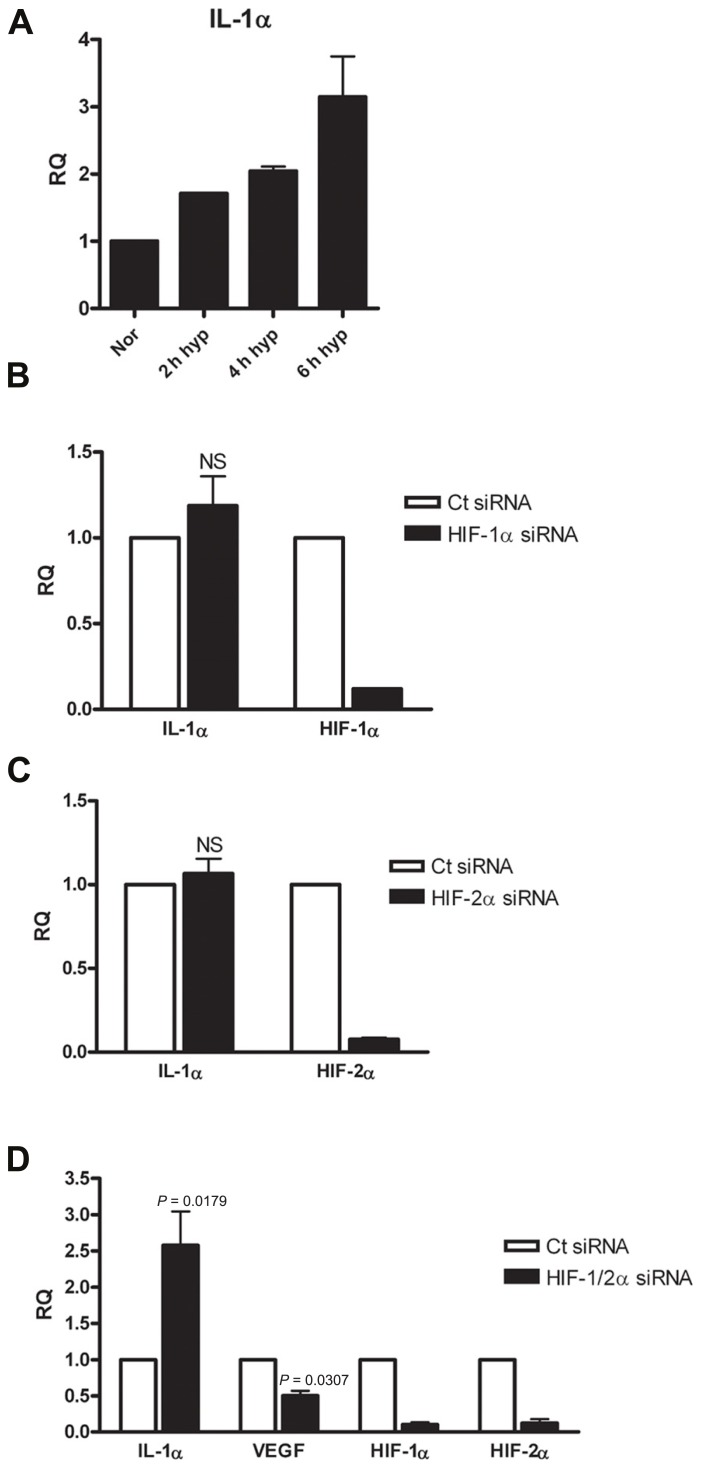
**Silencing HIFα proteins up-regulates IL-1α transcription in hypoxic WI-38 fibroblasts.(A)** WI-38 fibroblasts were cultured in either normoxic or hypoxic conditions for 2, 4, or 6 h, and were analyzed by qRT-PCR for RQ of IL-1α transcription. Graph represents the average ± SEM of two different experiments. RQ of IL-1α transcription in WI-38 cells transfected with HIF-1α **(B)**, HIF-2α **(C)** siRNA 24 h prior to 6 h culture in hypoxic conditions. Each graph represents mean ± SEM of three independent experiments. **(D)** Cells were transfected with both HIF-1α and HIF-2α mixed siRNA 24 h prior to hypoxia (6 h). Graph represents mean ± SEM of four independent experiments. *P* values of HIF-1/2α siRNA transfected cells vs. control (Ct) siRNA-transfected cells are annotated in the graphs.

Following the observation of decreased IL-1α transcription in A549 cells following HIFα silencing, we sought to confirm or decline whether the up-regulatory effect of HIFα factors on IL-1α expression is general observation or it is unique to this cell type, by testing different type of cells. Since fibroblasts and epithelial cells play different roles in inflammation and are known to interact and promote structural changes during inflammatory disease, such as asthma (Knight, 2001), we decided to test WI-38 fibroblasts cell line. These cells are capable of expressing IL-1α in similar way to the A549 cells (Figure S1 in Supplementary Material). The cells were incubated in hypoxic conditions for 2–6 h, time periods in which initial transcription alterations can be observed. Indeed, real-time PCR analyses showed that elevated levels of IL-1α mRNA correlated with the prolongation of hypoxia (**Figure 3A**). WI-38 fibroblast cells responded differently to HIFα silencing than A549 cells. While silencing HIF-1α or HIF-2α alone resulted in no significant change in levels of IL-1α mRNA (**Figures 3B**,**C**), silencing both HIF-1α and HIF-2α resulted in elevated levels of IL-1α transcription (**Figure 3D**). These data indicate that although IL-1α transcription is inhibited following HIFα silencing in A549 cells, fibroblasts respond in an opposite manner. To assure that this elevation was genuine and unaffected by the treatment itself, we ruled out differences in levels of cell death between A549 and WI-38 cells following the hypoxic culture (Figure S2 in Supplementary Material), and also examined VEGF levels, as VEGF is the most well-known pro-angiogenic factor controlled by HIFα factors. Indeed, while IL-1α mRNA levels increased following HIFα silencing, VEGF mRNA levels decreased as expected. These results indicate that in hypoxic fibroblasts, IL-1α transcription up-regulation is restrained by HIFα factors, and silencing these factors, for example for therapeutic intervention, can result in an increased inflammatory response, due to increased levels of IL-1α.

### OVEREXPRESSION OF HIFα FACTORS INCREASE IL-1α TRANSCRIPTION IN EPITHELIAL CELLS BUT NOT IN WI-38 FIBROBLASTS

**FIGURE 4:**
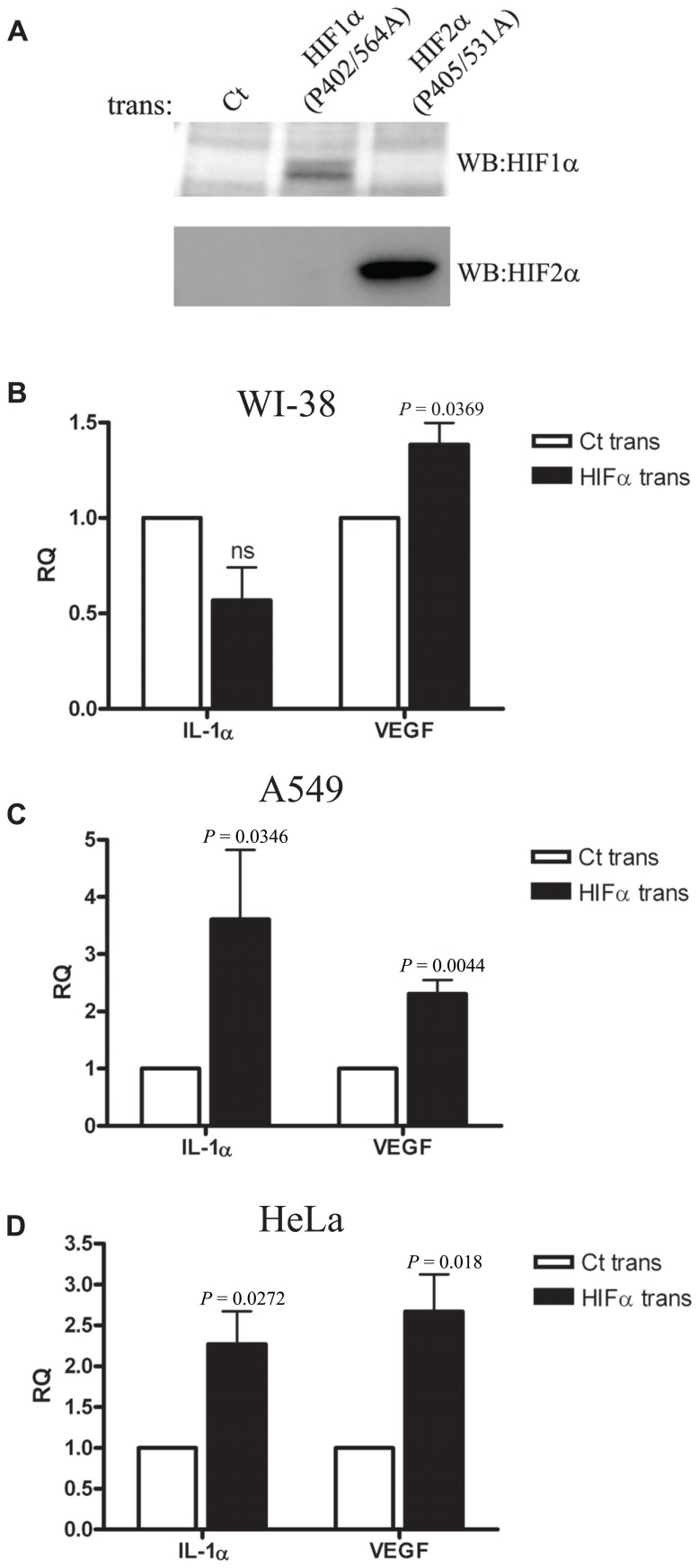
**Overexpression of HIFα proteins increases IL-1α transcription in A549 and HeLa cells but not in WI-38 fibroblasts.(A)** Western blot using anti-HIF-1α or anti-HIF-2α antibodies of nuclear fractions obtained from HEK-T293 cells transfected with the annotated vectors. WI-38 **(B)**, A549 **(C)**, or HeLa **(D)** cells were transfected with either control vector or with HIF-1α (P402A/P564A) and HIF-2α (P405A/P531A), and 24 h later were analyzed for IL-1α and VEGF RQ of mRNA levels by real-time PCR. Graphs represent mean ± SEM of four independent experiments for each cell type. *P* values of HIFα-transfected cells vs. control (Ct) transfected are annotated in the graphs.

We next decided to use a different approach to verify the results we obtained by siRNA silencing during hypoxia. We transfected WI-38 and A549 cells with plasmids encoding a proline to alanine muted form of HIF-1α and HIF-2α. These specific proline residues are hydroxylated under normal oxygen levels, and therefore mediate the degradation of the proteins by allowing the recognition of pVHL ubiquitin E3-ligase. In order to verify the stability of the muted proteins during normoxia, we obtained transfectant nuclear fractions and analyzed them by Western blot with anti-HIF-1α and anti-HIF-2α specific antibodies (**Figure 4A**). Indeed, increased levels of the proteins were obtained, as described before (Kondo et al., 2003). Next, we evaluated the effects of HIFα transfection on IL-1α levels in WI-38 and A549 cells. While higher mRNA levels of IL-1α were seen in A549 cells, IL-1α levels were not up-regulated in WI-38 cells, where, in fact, we noted a non-significant reduction in IL-1α levels (**Figures 4B**,**C**). Another epithelial cell line, the HeLa cell line, was transfected and evaluated for IL-1α by real-time PCR, and showed similar patterns to A549 cells, i.e., up-regulation of IL-1α transcription following an increase of HIFα factors by transfection (**Figure 4D**).

## DISCUSSION

Sterile inflammation is a process in which the immune system recognizes danger rather than stranger (Matzinger, 1994). IL-1RI was found to be the major innate receptor mediating the sterile inflammation response to dying cells (Chen et al., 2007). Moreover, a study made by our group shows that IL-1α, and not IL-1β, is the major mediator of the inflammatory response to necrotizing cells. In addition, IL-1α is retained together with the chromatin of apoptotic cells, preventing its release and the subsequent induction of inflammation. However, necrotic cells release IL-1α and recruit myeloid cells in an IL-1α-dependent manner (Cohen et al., 2010). Recently, we demonstrated that supernatants of hypoxic cells contain IL-1α, thus inducing sterile inflammation by recruiting neutrophils to the site of injury (Rider et al., 2011). The initial infiltration of neutrophils was dependent on IL-1α originating from necrotic cells. The cells were necrotic due to prolonged hypoxia, in which IL-1α was up-regulated, and finally released. As in the inflammatory process the first few hours are critical (Serhan and Savill, 2005) and include an influx of neutrophils, it was of interest to study the initial transcriptional response of IL-1α in cells undergoing hypoxic stress. Here, we show that IL-1α transcription increases during hypoxia in human cell lines. This allows the cells to adjust their transcriptome in order to alarm the surroundings of an approaching danger. When normoxic conditions are not restored, the cell will eventually die by necrosis. Some cells, such as keratinocytes, express large amounts of IL-1α under homeostatic conditions; nonetheless, IL-1α increases during hypoxia (Rider et al., 2011). Other cells should also exhibit increased expression of IL-1α immediately upon stress conditions, such as hypoxia. Indeed, both human epithelial cells and fibroblasts exhibit up-regulation of IL-1α during culture under hypoxic conditions. These data raised the question of whether HIFα factors were involved in IL-1α transcription. HIF-1α and HIF-2α were shown to up-regulate IL-1β in hypoxic macrophages (Fang et al., 2009). However, it was not evaluated whether these factors regulate also the expression of the alarmin cytokine IL-1α and whether it happens also in non-hematopoietic cells. Culturing the cells in a hypoxic chamber following siRNA silencing of HIFα factors, enabled us to determine that HIFα factors do regulate IL-1α transcription. In addition, we were able to distinguish between two different effects of HIFα regulation. In hypoxic A549 epithelial cells IL-1α is up-regulated by HIFα factors. However, in WI-38 fibroblasts, which originate in the lungs, as do A549 cells, HIFα factors regulate and restrain the transcription of IL-1α. This was verified using HIFα encoding vector transfections in normoxic cultures. The differential regulation of IL-1α transcription by HIFα proteins is still not clear. Further study is required in order to elucidate the opposing effect of these transcription factors on IL-1α in different types of cells. Indeed, in spite of numerous studies published concerning HIFα and inflammation, there have been no clear conclusions about the role of HIFα in inflammation. Injection of HIF-1α encoding vectors into mice showed a reduction in the IL-1α cytokine in splenocytes obtained one week after injection (Ben-Shoshan et al., 2009). Several other models of inflammation showed attenuating effects of HIFα (Kojima et al., 2007; Cummins et al., 2008; Kobayashi et al., 2012). However, HIFα factors in hypoxia were shown to cause an increase in cytokine levels, myeloid cell infiltration, and in the innate response (Nizet and Johnson, 2009). During inflammation, HIFα factors can play a significant role even without hypoxia, as HIFα proteins can be stabilized by NF-κB (Cummins et al., 2006; Rius et al., 2008). IL-1 signaling itself can increase the stability of HIFα and increase transcription of its target genes (Hellwig-Burgel et al., 1999). In addition, A549 epithelial cells, stimulated with IL-1β, were shown to increase the stability of the HIF-1α protein (Jung et al., 2003). Furthermore, viral infections of the lungs can stabilize HIFα proteins (Haeberle et al., 2008). However, when sterile inflammation occurs, hypoxia and not pathogens drives the inflammatory response (Nizet and Johnson, 2009). A growing number of studies show that IL-1α is a major mediator of sterile inflammation (Eigenbrod et al., 2008; Luheshi et al., 2009; Berda-Haddad et al., 2011; Lee et al., 2011; Rider et al., 2011; Gross et al., 2012; Norton et al., 2012). As such, its transcription during hypoxia has a special significance. Linking IL-1α up-regulation during hypoxia, a process which results in increased myeloid cell recruitment and HIFα transcription regulation raises the issue of the physiological relevance of these transcription factors in cases of sterile inflammation. Hypoxic factors are targeted in cancer therapy to use hypoxia-mediated cell death to kill cancerous cells; therefore, it is important to take into consideration that while pro-angiogenic factors, such as VEGF will be down-regulated, this treatment may either up- or down-regulate IL-1α, depending on the cell type. Elevated IL-1 levels can induce massive inflammation in the tissue; however, IL-1 itself can induce angiogenesis and compensate for the anti-angiogenic effects of HIFα inhibition, since IL-1 is an important mediator in angiogenesis (Carmi et al., 2009). Overall, our data suggest that HIFα factors can control the transcription of IL-1α during hypoxia. While transcription of IL-1α increased with HIFα in the lung epithelial cell line, A549, and in the HeLa cell line, IL-1α mRNA levels were attenuated by HIFα factors in lung fibroblasts. This is novel data concerning the induction of IL-1α-mediated sterile inflammation at the transcriptional level, in cells which are sensitive to hypoxic stress and are prone to necrosis.

## Conflict of Interest Statement

The authors declare that the research was conducted in the absence of any commercial or financial relationships that could be construed as a potential conflict of interest.

## SUPPLEMENTARY MATERIAL

Supplementary Material for this article can be found online at:http://www.frontiersin.org/Inflammation/10.3389/fimmu.2012.00290/abstract

**Figure S1 A549 and WI-38 cells express IL-1α**. Cells were stained by immunofluorescence for IL-1α and were analyzed by confocal microscopy (×600 magnification).

**Figure S2 Cell viability following 6 h hypoxia**. A549 and WI-38 cells were cultured with either normoxic or hypoxic conditions for 6 h. Cells were analyzed for annexin–PI by flow cytometer.
